# Recent Developments (After 2020) in Flow Cytometry Worldwide and Within China

**DOI:** 10.3390/bios15030156

**Published:** 2025-03-02

**Authors:** Xinyue Du, Xiao Chen, Chiyuan Gao, Junbo Wang, Xiaoye Huo, Jian Chen

**Affiliations:** 1 State Key Laboratory of Transducer Technology, Aerospace Information Research Institute, Chinese Academy of Sciences, Beijing 100190, China; duxinyue24@mails.ucas.ac.cn (X.D.); chenxiao20@mails.ucas.ac.cn (X.C.); gaochiyuan20@mails.ucas.ac.cn (C.G.); jbwang@mail.ie.ac.cn (J.W.); 2 School of Future Technology, University of Chinese Academy of Sciences, Beijing 100049, China; 3 School of Electronic, Electrical and Communication Engineering, University of Chinese Academy of Sciences, Beijing 100049, China

**Keywords:** flow cytometry, instrumentation, technical advances, research trends

## Abstract

This article reviews recent developments in flow cytometry that have a significant impact on both scientific research and clinical applications in the field of single-cell analysis, from the perspective of instrumentation and technical advances. As a starting point, this article investigates the latest state-of-the-art instruments of flow cytometry including different types in spectral, mass, imaging, nano, and label-free flow cytometry. A comparative analysis of the parameters and features of instruments from different companies elucidates the development trends in flow cytometry instrumentation. Following this, this article delves into cutting-edge technical advancements in flow cytometry. It summarizes the current research status of flow cytometry not only globally but also within China, highlighting emerging trends and innovations in the field. Finally, this article outlines future directions for the development of flow cytometry, indicating that each type of flow cytometry will follow its own trajectory toward achieving enhanced performance and broader applications in diverse fields.

## 1. Introduction

Flow cytometry (FCM) refers to a high-throughput technique of cell analysis that can simultaneously perform multi-parameter, high-precision, and fast quantitative analysis and sorting of single cells and biological micro particles in a fast linear flow state. As an enabling technology, flow cytometry can accurately monitor the numbers, phenotypes, and functions of blood, tumor, and stem cells, which plays irreplaceable roles in basic scientific research and the diagnosis and treatment of key diseases.

The first prototype of a flow cytometry instrument was reported in 1968 by Wolfgang Göhde at the University of Münste and commercialized by Partec (Munich, Germany) in collaboration with Phywe (Gottingen, Germany) AG in Göttingen. The first commercial instrument *Impulse Cytophotometer (ICP) 11* utilized light absorption as its detection method. Three years later, the Bio/Physics Systems (New York, NY, USA) developed *Cytofluorograph*, which was equipped with two lasers of 488 and 633 nm and capable of capturing forward scattered light and green and red fluorescence. In 1974, *FACS II* was developed by Becton Dickinson (Franklin Lakes, NJ, USA), enabling both optical measurements and droplet sorting. From the late 1970s, manufacturers of flow cytometry focused on the analysis performance of fluorescence detection. For example, *FACScan^TM^* of Becton Dickinson as well as EPICS C of Beckman Coulter could measure three colors simultaneously by including additional sources. Further improvements were witnessed by the simultaneous measurements of seven fluorescence parameters (*PAS-III* of Partec) and then fourteen fluorescence parameters (*LSR^TM^ II* of Becton Dickinson). Beckman Coulter has also launched other fluorescence flow cytometry products, including *CytoFLEX LX* in 2017, *DxFLEX* in 2018, and *Navios EX* in 2022. Currently, state-of-the-art fluorescence flow cytometry is equipped with 9 lasers and 50 detectors (*FACSymphony^TM^ A5* of BD Biosciences).

Within China, different commercial products are also being launched, which are represented by Challenbio. The *LongCyte™* series of Challenbio in 2022 can be equipped with three lasers and fourteen colors at most, and the latest updated *FongCyte™* series in 2024 can be configured with up to four lasers.

However, spectral overlap between fluorochromes in fluorescence flow cytometry can cause signal interference, which is referred to as spectral spillover. As a result, a variety of schemes of fluorescent compensation methods have been brought forward in order to address the issue of spectral spillover, obtaining some remarkable achievements. Initially, in 1977 Loken et al. employed an electronic cross-coupling apparatus to carry out immediate subtraction of the signals from both fluorescent yellow and rhodamine, achieving real-time fluorescent compensation [1]. Subsequently, in 1993 Bagwell and Adams expanded the field of fluorescent compensation by developing a data post-processing theoretical method that transcends hardware circuit limitations for real-time adjustments, allowing for the compensation of an unlimited number of fluorescence parameters based on the linear superposition of signals, exemplified by a three-color model [2], which is now a widely adopted protocol in multicolor fluorescence flow cytometry.

Within the last 20 years, the development of various types of flow cytometry has been witnessed, which can be classified into spectral, mass, imaging, nano, and label-free flow cytometry. Besides the instruments of flow cytometry, the cutting-edge technical developments in flow cytometry have been reported heavily, focusing on the higher throughput and higher dimension of imaging flow cytometry. Furthermore, the higher performance of fluorescence flow cytometry, mass flow cytometry, nano flow cytometry, and label-free flow cytometry are also under intensive study. This article takes a unique perspective by reviewing recent developments in flow cytometry from both instrumentation and technical advances, which hopefully can provide a comparative reference to the field of flow cytometry.

## 2. State-of-the-Art Instruments of Flow Cytometry

### 2.1. Spectral Flow Cytometry

Spectral detection was initially commercialized in imaging instruments and first applied to flow cytometry in the late 20th century. In 2007, Paul Robinson from Purdue University obtained a patent and was further authorized by Sony to commercialize the breakthrough spectrum flow cytometry of *SP6800*. In 2017, Cytek caught up with Aurora and sparked a wave of technological competition in spectral flow cytometry. Since then, the ranking of weapon spectra has undergone several changes, and major players have gradually entered the market such as *ID7000* of Sony in 2019, *Symphony A5* of Becton Dickinson in 2020, *Bigfoot* of ThermoFisher in 2020, and *FACSDiscover^TM^ S8* of Becton Dickinson (a combination of spectral and imaging flow cytometry) in 2022.

In 2024, both Agilent and ThermoFisher released their spectral flow cytometry: Agilent’s *Opteon* had a maximum configuration of 5 lasers and 73 detectors, while ThermoFisher’s *Attune^TM^ Xenith^TM^* had a maximum configuration of 5 lasers and 51 detectors. Meanwhile, Sony officially announced its full spectrum sorter of *FP7000* in 2023, where the configuration of the highest 6 lasers and 192 detectors still lead the way in the hardware of spectral flow cytometry. Encouragingly, Powclin from China also commercialized its own model of *SFLO*, which is equipped with 5 lasers and 64 detectors and starting to play more roles in spectral flow cytometry (see Table 1).

### 2.2. Mass Flow Cytometry

As a cytometer that utilizes mass spectrometry to perform multi-parameter detection of single cells, there are two main differences between mass and fluorescence flow cytometry. Firstly, the labeling system is different: the former mainly uses various fluorescent groups as labels for antibodies, while the latter uses various metal elements as labels. Secondly, the difference in detection systems is that the former uses lasers and photomultiplier tubes for detection, while the latter uses ICP mass spectrometry as the detection method. In comparison to flow cytometry of fluorescence, mass flow cytometry has key advantages such as a significantly increased number of detection channels, no interference between channels, no need to calculate compensation, and a large number of metal tags with an extremely low background [28,29].

The mass flow cytometry of *CyTOF* was initially marketed by DVS Sciences and currently *CyTOF XT* from the Standard BioTools is well regarded as the most advanced mass flow cytometer, capable of quantifying 135 channels simultaneously. Within China, Polaries, Powclin, and PLT Tech released their mass flow cytometry with models of *Starion^TM^* (140 channels), *MSFLO* (259 channels), and *PLT-MC601* (130 channels), respectively, becoming key players in mass flow cytometry (see Table 1).

In recent years, the rapid advancement and maturation of spectral flow cytometry have narrowed the applicability of mass cytometry, due to its high costs and lower throughput. However, mass cytometry remains indispensable for high-parameter detection with minimal spectral overlap.

### 2.3. Imaging Flow Cytometry

Imaging flow cytometry has added to the ability of cell detection based on microscopic imaging on the basis of fluorescence flow cytometry, aiming to capture the key information of cell morphologies. Currently, it is mainly used for analyzing the nuclear translocation of intracellular proteins, cell cycles, phagocytosis, cell death, subcellular structures, microbial analysis, and cell morphologies. The concept of imaging flow cytometry was proposed as early as the 1970s, and the first company to make commercial efforts, Amnis, was established in 1999 [30]. In 2005, the first generation of *ImageStream100* was launched, which was further acquired by Cytek.

From 2019 to 2024, the products of imaging flow cytometry were increased from three to at least seven–eight models, including *ImageStreamX Mark II* of Cytek, *FACSDiscover^TM^ S8* of Becton Dickinson, *Attune^TM^ CytPix^TM^* of ThermoFisher, *Verlo* of Nanocellect, *REM-I* of Deepcell, *CytoSense XR* of CytoBoy, and *FlowCam8000* of Yokogawa. Although the *ImageStream* series of Cytek can achieve clear cell images based on the 60× objective lens, the adoption of CCD for image capture means its analysis speed (5000 cells/s) does not match the speed of fluorescence flow cytometry. Moreover, due to the delays in taking cell images, this technology is not very compatible with cell sorting (see Table 1).

Thus, a series of low-latency and fast imaging techniques were pushed to the forefront by shaping the laser into a specific shape (linearly arranged multiple beams of laser) through optical devices and reconstructing it into an image using the differences in timing or frequency of the signals excited by different beams. For instance, Becton Dickinson’s *FACSDiscover^TM^ S8* utilizes a method similar to radiofrequency modulation in wireless communication to achieve multi-channel fluorescence high-speed imaging. It is the first commercial platform to combine fluorescence high-speed imaging with cell sorting, with a maximum imaging speed of 15,000 cells/s. In addition, the microfluidic manufacturer Nanocellect showcased its imaging flow cytometer of *Verlo*, which achieved cell imaging and sorting on a microfluidic platform where FPGA was used to achieve low-latency real-time image reconstruction and analysis.

Deepcell from the United States recently brought a new instrument that combined real-time analysis of unlabeled AI images with microfluidic sorting. This startup company from Stanford University adopted a highly disruptive analytical approach in its products, completely abandoning fluorescence detection and analyzing the morphological features of each cell into 115 dimensions [31]. It used AI algorithms and computer vision to deeply explore the biological significance behind each cell morphological feature, striving to establish the correlation between cell morphologies and lineage, functions, or states with applications in disease characterization, stem cell differentiation, and phenotype-based drug discovery.

### 2.4. Nano Flow Cytometry

Nano flow cytometry is a special improvement on the excitation light sheath fluid flow system, and detects based on the methods of traditional flow cytometry, being specifically designed for analyzing nanoscale particles including extracellular vesicles (exosomes), viruses, and liposomes [32].

Apogee Flow, a British company, was the first to commercialize nano flow cytometry, pushing the scattered light sensitivity to the order of 70 nm. NanoFCM in China pushed the scattered light sensitivity to within 30 nm at the single-molecule level. Furthermore, Beckman Coulter released its nano flow product of *CytoFLEX* Nano with a sensitivity of 40 nm polystyrene bead. These companies have accumulated numerous scientific projects and pharmaceutical enterprise users, significantly pushing forward the development of nano flow cytometry (see Table 1).

### 2.5. Label-Free Flow Cytometry

Similarly to the principle of “uncertainty” in physics, the uncertainty caused by disturbances in complex biological systems is even more elusive. Therefore, biological technicians also strive to minimize disturbances to their research subjects in their studies. The underlying logic of fluorescence flow cytometry is based on the recognition of cell-specific markers by fluorescence species, usually through antigen–antibody-specific binding or the specific binding of special fluorescein to specific molecules.

The process of staining or labeling, which typically involves fluorescent dyes or metal isotope tags, can introduce challenges and uncertainties in experiments. As a result, there has been growing interest in developing label-free technologies. However, the key issue is not the absence of labeling itself; rather, it lies in determining what exactly to measure and how to measure it accurately. The absence of labeling is only meaningful when cells can be identified and characterized with sufficient precision, ensuring that reliable measurements can still be made without the need for additional tags.

As to morphological features, strictly speaking all the types of imaging flow cytometry mentioned earlier can perform unlabeled image analysis, with Deepcell being a typical example. ThinkCyte from Japan uses *VisionSort* to achieve high-resolution morphological analysis of unlabeled single cells without image reconstruction. Based on lattice light source excitation and AI machine learning, it helped researchers explore sample types that fluorescence flow cytometry cannot handle.

In terms of electrical characteristics, various macromolecules that make up cells have different electrical characteristics. CytoRecovery showcased a device of *CytoR1* for the sorting of specific cell populations based on cellular electrical characteristics. Combined with microfluidic chips and proper controls of electronic signals, T and B cells were separated through electrical control, achieving low damage and label-free cell processing. The Swiss company Amphasys showcased its impedance flow cytometry of *Ampha X30*, which used particle impedance as the detection index and is mainly applied in areas such as plant pollen and environmental and industrial microbial detection (see Table 1).

## 3. Cutting-Edge Developments in Flow Cytometry

### 3.1. Cutting-Edge Developments in Flow Cytometry: Worldwide

**Figure 1:**
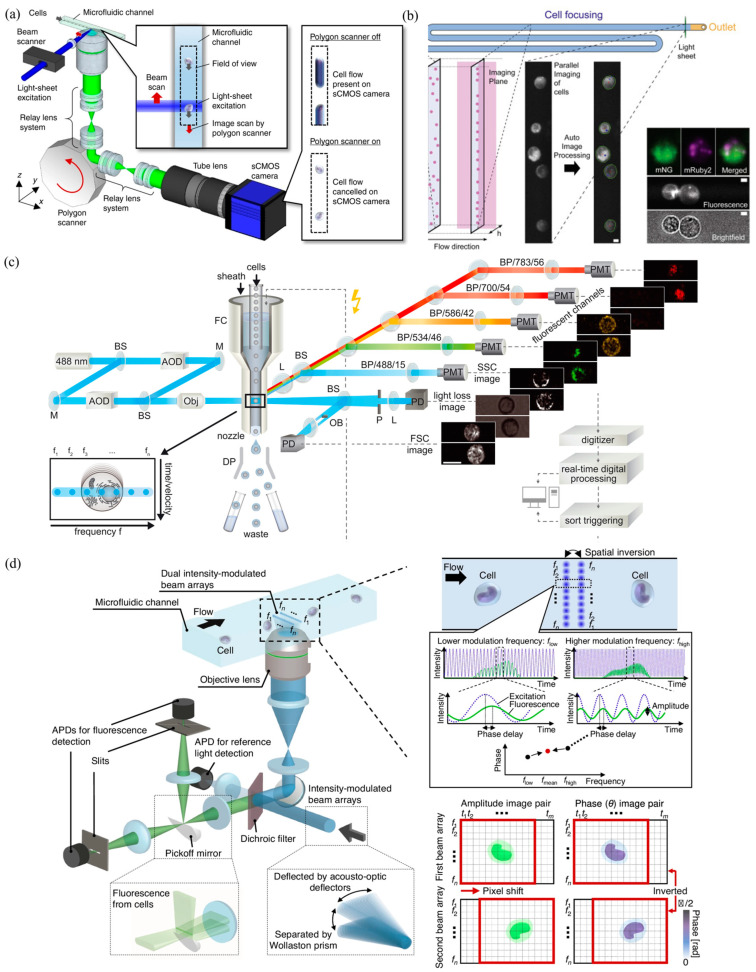
High-throughput imaging flow cytometry: (**a**) Imaging flow cytometry with the capability of virtual-freezing fluorescent images with a throughput >10,000 cells/s (Figure permission from [33]); (**b**) Microfluidic imaging flow cytometry coupled with a stroboscopic light sheet with a throughput of 60,000 cells/s (Figure permission from [34]); (**c**) Imaging flow cytometry based on radiofrequency-tagged emission with a throughput of 15,000 cells/s (Figure permission from [35]); (**d**) Fluorescence lifetime imaging flow cytometry based on dual intensity-modulated continuous-wave beam arrays with a throughput >10,000 cells/s (Figure permission from [36]).

Within the field of flow cytometry, the current focus is definitely imaging flow cytometry. Firstly, several key studies were conducted to improve the throughput of imaging flow cytometry. As shown in Figure 1a, Goda et al. [33] presented an optomechanical imaging method to virtually “freeze” the rapid movement of travelling cell cameras by rotating the polygon scanner in the opposite direction, realizing a throughput >10,000 cells/s. Furthermore, deMello et al. presented an imaging flow cytometry of microfluidics where a viscoelastic carrier fluid enabled the movement of accurately focused cells to the central plane within the microfluidic cuvette, enabling the imaging of multiple cells in one shot, producing a throughput >400,000 cells/s [34] (see Figure 1b).

Aiming to further enhance the capture rate of imaging flow cytometry, CCDs were replaced by PMTs with radiofrequency-tagged emission utilized. As shown in Figure 1c, the approach of radiofrequency-tagged emission was leveraged to generate arrayed laser spots with unique radiofrequency modulation, which excited fluorescence and other scattered lights from travelling cells. Emitted lights with modulation were captured and further analyzed by FPGAS, allowing image analysis in a real-time manner, producing a throughput of 15,000 cells/s [35]. A similar idea was utilized to realize high-speed imaging of fluorescence lifetime imaging flow cytometry where beam arrays with both intensity and frequency modulation were incorporated with fluorescence excitation and capture, with a throughput of 10,000 cells/s [36] (see Figure 1d).

The development of imaging flow cytometry is trending rapidly toward high-speed imaging. However, the critical question remains unclear regarding its practical applicability in clinics. Hopefully, the significantly increased imaging speed can truly enable clinical applications, such as achieving three-part or five-part leukocyte differentials.

**Figure 2:**
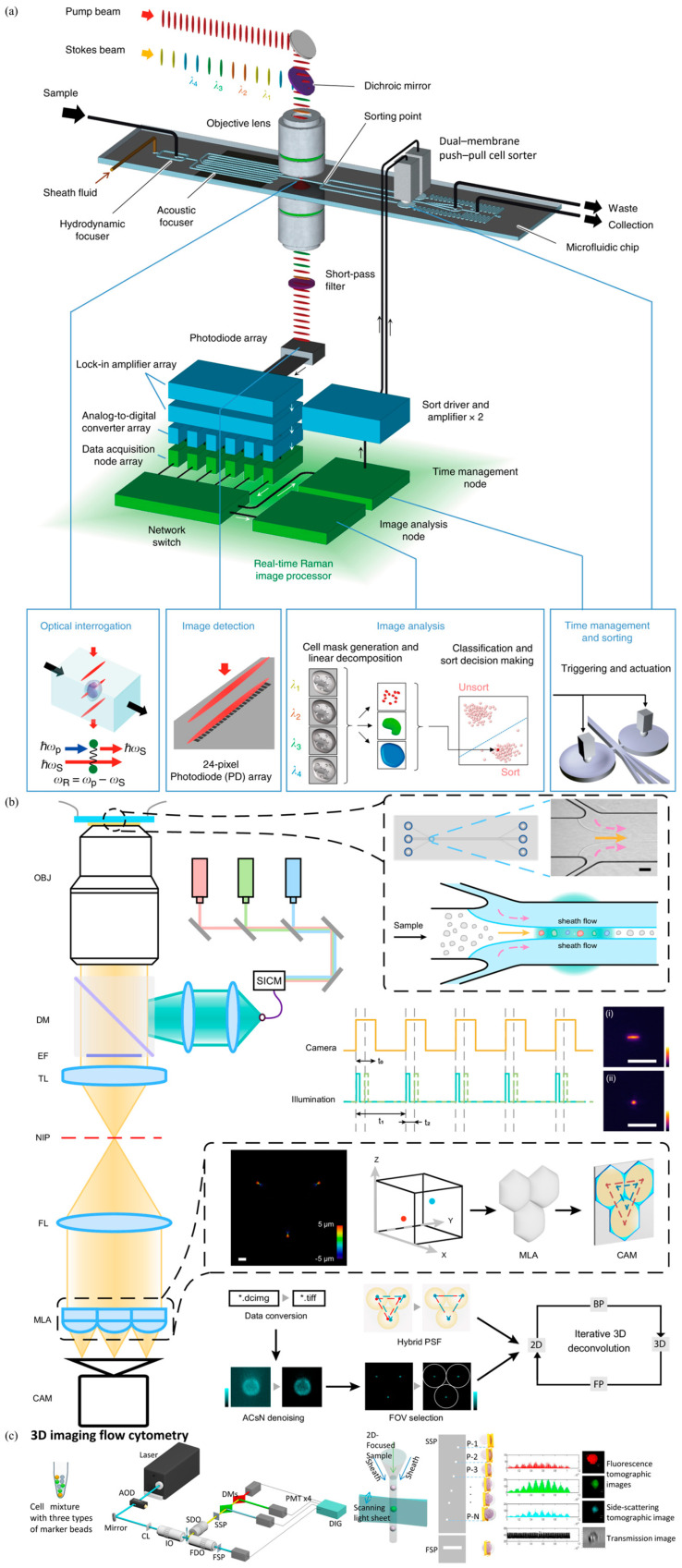
High-dimension imaging flow cytometry: (**a**) Raman imaging flow cytometry where scattering images of stimulated Raman spectroscopy could be captured rapidly (Figure permission from [37]); (**b**) Light-field flow cytometer for high-speed 3D analysis of subcellular morphologies (Figure permission from [38]); (**c**) 3D imaging flow cytometry using a scanning light sheet and spatial filter, aligning pinholes with cell flow to produce 3D tomographic images from fluorescently labeled cells (Figure permission from [39]).

Besides higher throughputs, several breakthroughs were achieved recently in the field of higher dimensions of imaging flow cytometry. For instance, Goda et al. reported high-speed Raman imaging based on a unique laser source with a switchangle wavelength and pulse-pair resolution, enabling a throughput of up to ~100 cells/s (see Figure 2a) [37]. Another breakthrough in the field of higher dimensions of imaging flow cytometry was to convert traditional 2D images into informative 3D images. Jia et al. reported a light-field flow cytometer designed for the analysis of various 3D morphologies of travelling cells in multiple spatial dimensions at high speeds, in which the native fluorescent image was transformed into elemental light-field images via a hexagonal microlens array, which were regarded as a convolution between the light-field point-spread function and the multidimensional spatial morphology of cells and utilized to construct 3D images of travelling cells (Figure 2b) [38]. Lo et al. also reported a 3D imaging flow cytometry based on a scanning light sheet coupled with a spatial filter where a customized spatially positioned pinhole array was adopted with the cell flow, producing the 3D tomographic images reconstructed from the emitted light of fluorescently labeled cells detected by photomultiplier tubes (Figure 2c) [39]. Of course, artificial intelligence was also included in image flow cytometry, where, for instance, a protocol of Deepometry with both strongly and weakly supervised cell classification was developed recently, which can work properly for both training from scratch and based on a pretrained model [40].

Although artificial intelligence has enhanced its capabilities, the imaging quality of multidimensional imaging flow cytometry remains generally lower than that of confocal microscopy, primarily due to limitations in resolution and optical sectioning capabilities. Consequently, the critical challenge for multidimensional imaging flow cytometry lies in its ability to meet the demands of clinical applications.

**Figure 3:**
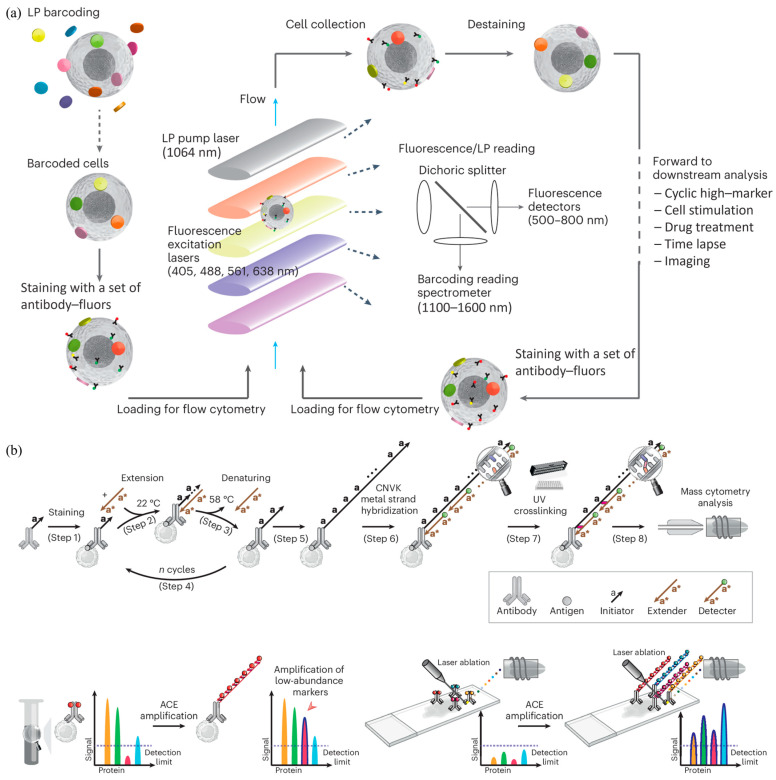
High-performance fluorescence and mass flow cytometry: (**a**) High-content fluorescence flow cytometry with barcoded single cells based on spectral encoding (Figure permission from [41]); (**b**) High-resolution mass flow cytometry where cycle extension was included for signal amplification (Figure permission from [42]).

The higher performance of fluorescence and mass flow cytometry were also reported recently. More specifically, Yun et al. reported a high-content flow cytometry where individual cells with their own spectral encoding were allowed to travel through fluorescence flow cytometry multiple times with corresponding fluorescent labels measured [41] (see Figure 3a). By using blood cells of live humans, it was demonstrated that the approach realized time-resolved measurement of the same leukocytes before and after chemical stimulations by forcing these cells to travel through the detection cuvette. In addition, Yin et al. reported high-resolution mass flow cytometry where cyclic extension was used for signal amplification [42] (see Figure 3b). As a functional demonstration, this approach was used in tissue imaging of mass cytometry to successfully profile kidney tissues with low-expression cytokines.

### 3.2. Cutting-Edge Developments in Flow Cytometry Within China

**Figure 4:**
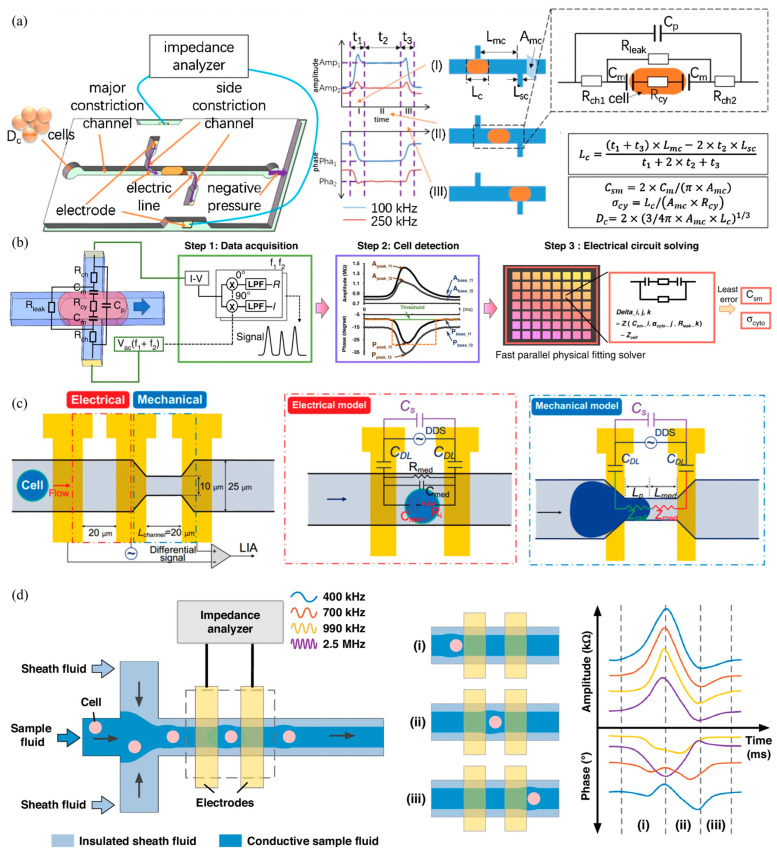
Key developments in impedance flow cytometry of constrictional microchannels within China: (**a**) Electrical flow cytometry incorporated with constrictional microchannels (Figure permission from [43]); (**b**) Real-time impedance flow cytometry utilizing a fast parallel fitting solver for constrictional microchannels (Figure permission from [44]); (**c**) Predicting single-cell mechanical properties via impedance profiles in constrictional microchannels (Figure permission from [45]); (**d**) Virtual constrictional microchannels using cross-flow sheath flow for high-sensitivity impedance measurements and accurate leukocyte classification (Figure permission from [46]).

As to cutting-edge developments in flow cytometry within China, a key research direction is the development of high-sensitivity label-free impedance flow cytometry. As shown in Figure 4a, Chen’s group from the State Key Laboratory of Transducer Technology pioneered the development of electrical flow cytometry incorporated with constrictional microchannels, in which electric lines and cells under measurements were effectively constricted to significantly improve the signal–noise ratio of single-cell impedance variations [43]. Further technical improvements were witnessed by Huang’s group from the Chinese Academy of Sciences where a physical solver with a parallel fitting solver was included to realize impedance flow cytometry of constrictional microchannels in a real-time manner (see Figure 4b) [44] as well as by Wang’s group from the Tsinghua University where impedance profiles of constrictional microchannels were also used to predict mechanical properties of single cells (see Figure 4c) [45]. In order to deal with the potential concern of channel blockage, the concept of a virtual constrictional microchannel was recently developed, where cross-flow sheath flow was utilized to confine single cells within the conductive flow channel for high-sensitivity impedance measurements, enabling high classification accuracies of leukocyte subtypes without clogging [46,47] (see Figure 4d). The clinical application of impedance flow cytometry with constrictional channels is currently limited due to the issue of channel blockage. To enable its practical use in clinical settings, future research should prioritize solving the blockage problem.

**Figure 5:**
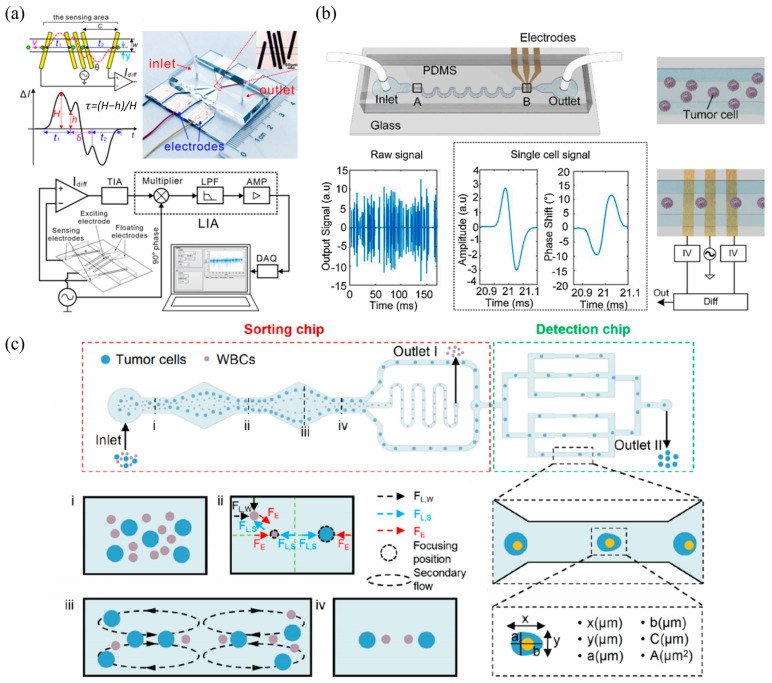
Key development of label-free flow cytometry based on fine-tuned geometrical dimensions within China: (**a**) Impedance flow cytometry with floating electrodes enabling 3D position determination of single cells (Figure permission from [48]); (**b**) Asymmetric serpentine structure enabling precise cell positioning and reduced impedance fluctuations (Figure permission from [50]); (**c**) Deformability flow cytometry with viscoelastic fluid flow capable of high-throughput mechanical characterization and sorting of single cells (Figure per-mission from [51]).

Besides constrictional microchannels, fine-tuned geometrical dimensions of label-free flow cytometry based on microfabrication were also intensively conducted by scholars from China. For instance, Wang’s group from Tsinghua University developed an impedance flow cytometry incorporated with floating microelectrodes to determine single-cell geometrical positions by analyzing both induced currents from floating microelectrodes and travelling currents of conductive microelectrodes for a travelling cell (see Figure 5a) [48]. Meanwhile, Xiang’s group from the Southeast University integrated a microstructure of asymmetric serpentine to realize 3D focusing of a travelling cell based on elasto–inertial forces and thus fluctuations in single-cell impendence variations could be avoided (see Figure 5b) [49,50]. Furthermore, the same group reported deformability flow cytometry, where viscoelastic forces were included for mechanical characterization and sorting of tumor cells as a demonstration of fine-tuned microchannels in flow cytometry (see Figure 5c) [51].

**Figure 6:**
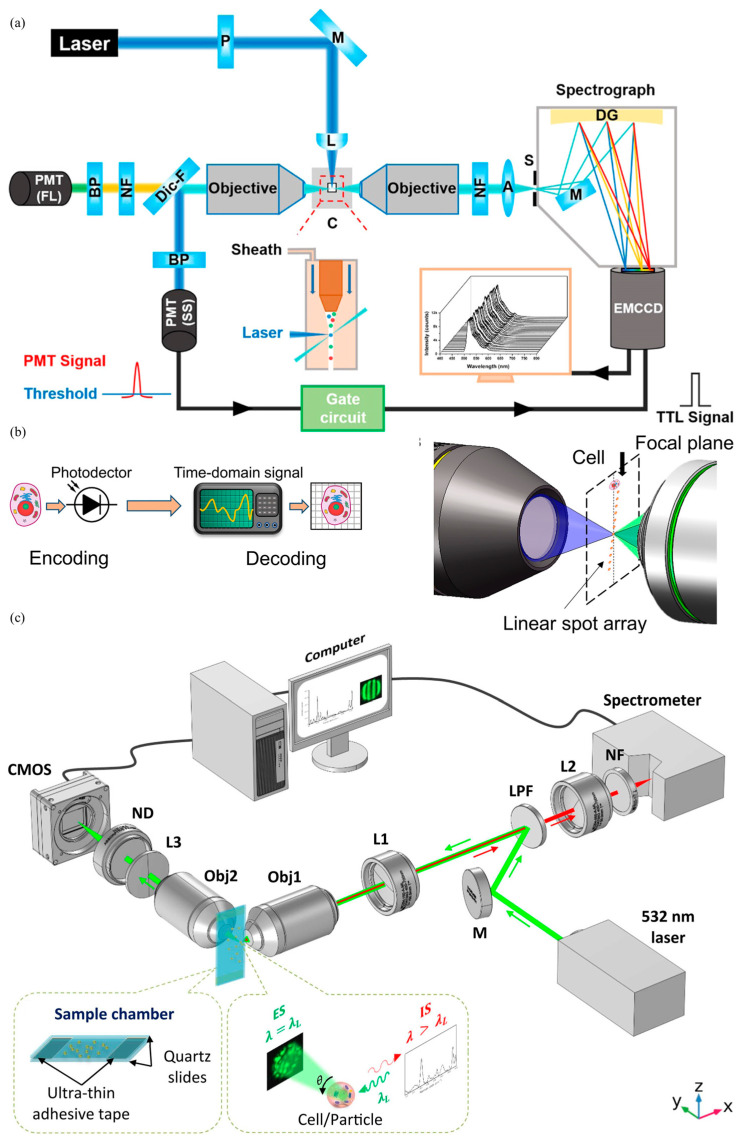
Key developments in high-performance mass and imaging flow cytometry within China: (**a**) Spectral nano flow cytometry with enhanced sensitivity in side scattering and fluorescence detection (Figure permission from [52]); (**b**) Imaging flow cytometry incorporated with a linear spot array for excitation to improve scalability (Figure permission from [53]); (**c**) Scattering image–spectro microscopy for simultaneous side elastic scattering and Raman spectrum measurements (Figure permission from [54]).

As to the development of other types of flow cytometry with labeling, Yan’s group from the Xiamen University reported spectral nano flow cytometry, where a spectral unit was integrated to home-developed nano flow cytometry, enabling sensitivity improvements from both scattering lights and fluorescence (see Figure 6a) [52]. In addition, Zhao’s group from the Huazhong University of Science and Technology developed imaging flow cytometry incorporated with a linear spot array for excitation, significantly extending scalability (see Figure 6b) [53]. Furthermore, Su’s group from Shandong University integrated elastic and Raman scattering based on a single-wavelength excitation to effectively investigate cell samples in a label-free manner (see Figure 6c) [54].

## 4. Future Developments in Flow Cytometry

With the full bloom of spectral flow cytometry, it can be foreseen that high-end multicolor (20+color) fluorescence flow cytometry may be replaced in the near future and the living space of mass flow cytometry will also be further squeezed. The imaging flow cytometry has surpassed its infancy and is moving toward a period of rapid growth, which will continue to see continuous expansion and accumulation of applications. In the future, it is foreseeable that imaging flow cytometry is moving toward deep integration, and will jointly reproduce more and more products with different forms.

Following the waves of exosomes, gene therapy, and mRNA vaccines, nano flow cytometry has also passed the initial stage of questioning and begun to be widely adopted and focused on. As a branch growing from the backbone of flow cytometry, nano flow cytometry is welcoming spring in its own scenery. The biggest challenge facing the instrumentation of unlabeled flow cytometry is how to establish a connection between the above cell characteristics and the cell identification system (biological significance) commonly used by biologists. If this goal can be achieved, it may lead methodologies of flow cytometry to a new paradigm.

## Figures and Tables

**Table 1:** A summary of the state-of-the-art instruments of flow cytometry, worldwide and within China.

Instrument	Manufacturer	Classification	Parameter
FACSymphony™ A5 [3]	Becton Dickinson	Fluorescence Flow Cytometry	9 lasers and 50 detectors
CytoFLEX LX [4]	Beckman Coulter	Fluorescence Flow Cytometry	6 lasers and 21 detectors
DxFLEX [5]	Beckman Coulter	Fluorescence Flow Cytometry	3 lasers and 13 detectors
Navios EX [6]	Beckman Coulter	Fluorescence Flow Cytometry	3 lasers and 12 detectors
LongCyte™ [7]	Challenbio@China	Fluorescence Flow Cytometry	3 lasers and 14 detectors
FongCyte™ S [8]	Challenbio@China	Fluorescence Flow Cytometry	4 lasers and 14 detectors
FP7000 [9]	Sony	Spectral Flow Cytometry	6 lasers and 182 detectors
FACSDiscover™ S8 [10]	Becton Dickinson	Spectral and Imaging Flow Cytometry	5 lasers and 86 detectors including 6 imaging detectors
Aurora^TM^ CS [11]	Cytek	Spectral Flow Cytometry	5 lasers and 67 detectors
NovoCyte Opteon [12]	Agilent	Spectral Flow Cytometry	5 lasers and 73 detectors
Attune™ Xenith™ [13]	ThermoFisher	Spectral Flow Cytometry	6 lasers and 51 detectors
SFLO [14]	Powclin@China	Spectral Flow Cytometry	5 lasers and 64 detectors
CyTOF XT [15]	Standard BioTools	Mass Flow Cytometry	135 channels
Lunarion [16]	Polaris@China	Mass Flow Cytometry	140 channels
MSFLO [14]	Powclin@China	Mass Flow Cytometry	259 channels
PLT-MC601 [17]	PLT Tech@China	Mass Flow Cytometry	130 channels
Amnis ImageStreamX Mark II [18]	Cytek	Imaging Flow Cytometry	5000 cells/s
Attune™ CytPix™ [19]	ThermoFisher	Imaging Flow Cytometry	5000 cells/s
Verlo [20]	Nanocellect	Imaging Flow Cytometry	2000 cells/s
REM-I [21]	Deepcell	Imaging Flow Cytometry	1000 cells/s
ApogeeFlow Cytometers [22]	Apogee Flow	Nano Flow Cytometry	100 nm silica/70 nm polystyrene bead
Flow Nanoanalyzer [23]	NanoFCM@China	Nano Flow Cytometry	40 nm exosome and virus
CytoFLEX Nano [24]	Beckman Coulter	Nano Flow Cytometry	40 nm polystyrene bead
VisionSort [25]	ThinkCyte	Label-Free (morphological) Flow Cytometry	3000 cells/s
CytoR1 [26]	CytoRecovery	Label-Free (morphological) Flow Cytometry	
Ampha X30 [27]	Amphasys	Label-Free (impedance) Flow Cytometry	5000 cells/s
